# Effect of Genetic Diversity in Swine Leukocyte Antigen-*DRA* Gene on Piglet Diarrhea

**DOI:** 10.3390/genes7070036

**Published:** 2016-07-15

**Authors:** Xiaoyu Huang, Qiaoli Yang, Junhu Yuan, Lixia Liu, Wenyang Sun, Yingdi Jiang, Shengguo Zhao, Shengwei Zhang, Wangzhou Huang, Shuangbao Gun

**Affiliations:** 1 College of Animal Science and Technology, Gansu Agricultural University, Lanzhou 730070, China; huanghxy100@163.com (X.H.); yangql0112@163.com (Q.Y.); sun_china@outlook.com (W.S.); 15600660313@163.com (Y.J.); zhaosg@gsau.edu.cn (S.Z.); zhangsw66@sina.cn (S.Z.); huangwangz@126.com (W.H.); 2 Ruzhou Animal Husbandry Bureau, Ruzhou 467500, China; 18893703880@163.com; 3 College of Life Science and Engineering, Northwest University for Nationalities, Lanzhou 730070, China; skllx@xbmu.edu.cn; 4 Gansu Research Center for Swine Production Engineering and Technology, Lanzhou 730070, China

**Keywords:** *SLA-DRA*, genetic diversity, PCR-SSCP, diarrhea, swine

## Abstract

The swine leukocyte antigens (SLAs) are the multigene families related to immune responses. Little is known about the effect of the *DRA* gene on diarrheal disease. This study reported the genetic diversity of the *DRA* gene in exons 1, 3 and 4 in 290 Chinese Yantai black pigs. No variation was identified in exon 3. In exon 1, three genotypes and two alleles were identified, generated by two single nucleotide polymorphisms (SNPs). In exon 4, there were eight genotypes and five alleles containing seven SNPs were detected with four SNPs being novel SNPs. The low polymorphism found in swine *DRA* is consistent with the concept that the *DRA* gene is highly conserved among all mammalian species. Statistical analyses indicated that the genotypes of exon 1 were not significantly associated with piglet diarrhea (*p* *>* 0.05); however, genotypes *C_4_C_4_* (1.80 ± 0.33) and *A_4_E_4_* (1.66 ± 0.25) of exon 4 were significantly susceptible to diarrhea (*p* *<* 0.01). These indicate that the particular genotypes of the *DRA* gene are susceptible to diarrheal disease, which provides valuable information for disease-resistance breeding in swine.

## 1. Introduction

The swine major histocompatibility complex (MHC) is a large multigene family that encodes the cell surface membrane-bound glycoproteins known as swine leukocyte antigen (SLA) molecules [1,2]. SLA molecules play a vital role both in acquired immune response to various infectious agents and vaccinations, and in reproductive performance and production traits [1]. Mapped to chromosome 7 spanning the centromere, SLA molecules are organized by SLA class I, II and III regions. Class II antigens are heterodimers consisted of 34 kDa α chain non-covalently bound to 29 kDa β chain, class II antigens mainly present self and non-self antigen peptides to circulating CD^4+^ helper T cells in adaptive immune responses and are split into *SLA-DR*, -*DQ*, -*DM* and -*DO* genes [3]. The *DR* and *DQ* genes are uniquely expressed on the surface of professional antigen presenting cells, and form a peptide-binding groove. Both *DR* (*DRA* and *DRB1*) and *DQ* (*DQA* and *DQB1*) genes are comprised of four exons with exon 1 encoding the leader sequence, exon 2 and 3 encoding the corresponding extracellular α1 and α2 functional domains, and exon 4 encoding transmembrane and cytoplasmic domains [1,4].

The *MHC-DRA* gene is considered to be conserved in mammalian species, such as the *DRA* gene across the cattle species [5,6]. However, the gene has been cloned and sequenced in many other species with some variations reported: Chinese rhesus macaques [7], donkeys [8], equine [9] and sheep [10] in which allelic diversity of *DRA* is found to be higher than that reported in other vertebrates. Currently, 13 *DRA* alleles of swine have been recorded in the Immune Polymorphism Database (IPD)-SLA (http://www.ebi.ac.uk/ipd/mhc/sla/nomenclature.html), while only 6 *DRA* alleles of cattle and 11 *DRA* alleles of ovine are recorded. A relatively high level of variation appears in *DRA* gene of pigs.

MHC plays a crucial role in modulating and maintaining overall adaptive immunologic resistance to pathogens and has become the hotspot for the associations between immune properties and diseases in vertebrates, such as human infectious disease [11], sheep cystic echinococcosis [12], and chicken mark’ disease [13]. Research indicates that the *BoLA-DRB3* gene plays a role in controlling the proviral load in cattle [14], and in sheep, particular *DQA2* alleles are associated with susceptibility to gastrointestinal nematodes parasites [15] and footrot disease [16].

Studies in pigs have indicated that SLA molecules have some implications and association with immune diseases, such as diarrhea [17,18,19,20,21], foot and mouth disease virus (FMDV) [22], malignant melanoma of skin [23], porcine reproductive and respiratory syndrome virus (PRRSV) [24], porcine pseudorabies virus (PRV) [25] and xenotransplantation [26]. In our previous studies, we investigated the genetic diversities of *DQA* and of *DRA* loci and reported their association with piglet diarrhea in introduced pig breeds (Large White, Landrace and Duroc) and Chinese native breeds (Bamei, Juema, and Gansu black pig) [17,18,19,20,21]. Further study on SLA polymorphisms in other breeds will enrich our knowledge on the swine MHC family.

The importance of pigs in animal husbandry development and biomedical and veterinary research has significantly increased. Sucking piglet diarrhea is a common destructive disease with high morbidity and mortality, and poses a serious economic impact to the swine industry worldwide. Diarrhea is reported to have a mortality rate of 15%–25% and a death rate of up to 49.11% of the mortality toll in piglets [27]. Diarrhea is mainly caused by genetic factors and poor management [28,29], but the major factor is the host genetics related to the immune relevant genes, such as SLA molecules [30]. Looking for the association between SLA molecular characteristics and disease resistances is important to swine disease-resistant breeding [31,32].

Yantai black pigs are a local gray skin breed in Shandong Province, China. This breed has many resilient characteristics, such as high growth performance, resistance of forage and strong disease resistance. Yantai black pigs have become a recognized healthy and safe meat resource and make great contribution to the economic development of the pig industry in China. Currently, relatively little is known about the *DRA* variation in this pig breed.

We have previously identified variation in exon 2 of *DRA* in Yantai black pigs and reported its association with diarrhea [20]. In this study, we investigated genetic variation of *DRA* in exons 1, 3 and 4, and the association between variation in the *DRA* gene and piglet diarrhea. Identification of SNPs in the *DRA* gene will enrich the knowledge on SLA diversity and facilitate the selection of potential candidate genes associated with piglet diarrhea, which will be eventually applied to cultivate a highly diarrhea-resistant breed in the swine industry.

## 2. Materials and Methods

### 2.1. Samples Collection and DNA Extraction

All research involving animals was carried out in accordance with Chinese criteria for animal welfare and the experimental protocol approved by the Animal Care and Use Committee of the Institute of Subtropical Agriculture, Chinese Academy of Sciences.

Ear tissue samples were collected from 290 Yantai black pig piglets with clear genealogy in Honggu Region, Gansu Province, China. The samples were preserved in tubes with 5 mL alcohol, stored on ice and transported to the laboratory for DNA isolation. All piglets were progeny from 31 sows during June–October 2013, and were conducted under the uniform fodder, feeding conditions and disease control, which was in accordance with the guide for Chinese Feeding Standard of Swine approved by Ministry of Agriculture Feed Industry Center, China Agricultural University (Beijing, China). Data collection on piglet diarrhea was performed using the method of Yang et al. [18]. Genomic DNA was extracted using the standard phenol-chloroform extraction procedure [33] and stored at −20 °C.

### 2.2. PCR Amplification and Single-Stranded Conformational Polymorphism (SSCP) Analysis

Three pairs of PCR primers were designed to amplify *DRA* exons 1, 3 and 4 fragments, based on the reference *DRA* sequence (GenBank accession No. AY303990) using the Primer 5.0 software (Premier Biosoft International, Palo Alto, CA, USA). Primer sequences are shown in Table 1. The sequences amplified covered the whole exons and parts of introns.

PCR amplifications were performed in a 25 μL final volume consisting of 2.5 μL 10 × PCR buffer (including 15 mM Mg^2+^), 1 μL genomic DNA, 0.5 μL each primer (10 μM), 1 μL dNTPs (2.5 mM each), 0.5 μL Taq DNA polymerase (5 units/μL) (TaKaRa, Japan), and ddH_2_O made up the final volume. PCR amplifications consisted of 94 °C for 3 min, followed by 35 cycles at 94 °C for 30 s, 30 s at annealing temperature as shown in Table 1, 72 °C for 30 s, and final extension at 72 °C for 10 min.

PCR products were detected by 2% agarose gel electrophoresis (Biowest Agarose, Nuaillé, France) using 1 × TBE buffer (89 mM Tris, 89 mM boric acid, 2 mM Na2EDTA, pH 8.0). Gels were stained with nucleic acid fuel (BioTeKe, Beijing, China) and photographed under UV light (1000× Press, Syngene, Cambridge, UK).

Polymorphisms of *DRA* exons 1, 3 and 4 were analyzed by single-strand conformation polymorphism (SSCP) [34]. A 3 μL aliquot of each amplimer was mixed with 7 μL denaturing solution (98% formamide, 0.025% bromophenol blue, 0.025% xylene cyanol, 20 mM EDTA, pH 8.0). After heat denaturation at 98 °C for 10 min, the samples were immediately chilled on ice for 10 min to prevent heteroduplex formation and then loaded onto 17 cm × 17 cm, 10% or 12% acrylamide:bis-acrylamide gels (29:1 or 39:1 acrylamide:bis-acrylamide). Electrophoresis was performed in 1 × TBE under the conditions described in Table 1. Gels were visualized by silver staining after electrophoresis according to the method of Byun et al. [35].

### 2.3. Cloning and Sequencing

Different PCR amplimers of each SSCP pattern were selected for purification using the DNA Fragment Quick Recover Kit (Tiangen, Beijing, China). Homozygous samples from PCR-SSCP analysis were directly sequenced, and heterozygous samples were ligated into the pMD19-T vector (TaKaRa, Shiga, Japan). The ligation mixture was used to transform competent *E. coli DH5α* cells. In total, 8–10 insert-positive clones obtained from each transformation were selected and incubated in 4 mL LB broth overnight in a thermostat water bath at 37 °C in a Forma orbital shaker (220 rpm) (Thermo, Waltham, MA, USA). All positive clones were screened using the PCR-SSCP approach [34], and only positive clones whose SSCP results matched the original genomic DNA SSCP results were selected and sequenced in the forward and reverse direction by Sangon Biotech Co., Ltd. (Shanghai, China). Briefly, a band corresponding to the allele was excised as a gel slice from the polyacrylamide gel, purified, cloned and then used as a template for re-amplification with the original primers. This second amplicon was re-run on the gel and then sequenced.

### 2.4. Sequence and Statistical Analysis

Nucleotide sequences and amino acid alignments of the *DRA* gene were analyzed by MEGA 6.0 software (CEMI, Tempe, AZ, USA) [36]. The identified SNPs were compared with the swine sequence available in NCBI BLAST. ExPASy web server (Swiss Institute of Bioinformatics, Geneva, Switzerland) was used to translate nucleotides to amino acids.

The frequency distribution of genotypes and alleles, the number of effective alleles (*A_E_*), observed heterozygosity (*oHet*) and expected heterozygosity (*eHet*) were estimated using POPGENE 3.2 software (Molecular Biology and Biotechnology Centre, University of Alberta, Edmonton, AB, Canada). The polymorphism information content (*PIC*) was calculated using Botstein’s method [37]. The LD value was calculated using SHEsis software platform (Bio**–**X institutes of Shanghai Jiaotong University, Shanghai, China).

Associations between genotypes and piglet diarrhea were estimated using the Generalized Linear Model (GLM) in the SPSS 17.0 software (SPSS Inc., Chicago, IL, USA) as follows:

Y_ijk_ = μ + B_i_+ G_j_ + S_k_ + e_ijk_ (1)

where Y_ijk_ = the observed piglet diarrhea score, μ = the mean of the population, B_i_ = the effect of the season, S_k_ = the fixed effect of sex, G_j_ = the effect of the genotype, and e_ijk_ = the random error.

## 3. Results

### 3.1. SSCP Analysis of SLA-DRA Gene Exons

**Figure 1:**
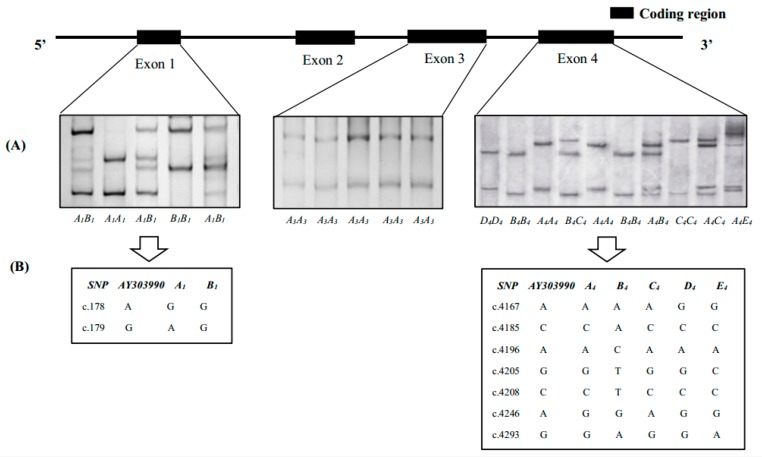
The SSCP analysis results and the single nucleotide variations identified in the *SLA-DRA* gene of Yantai black pigs. Unique PCR-SSCP results (**A**) representing different genotypes were detected in exon 1, 3 and 4. The coordinates of SNPs were annotated (**B**) based on the swine *SLA-DRA* reference sequence (accession no: AY303990) in GenBank.

Three distinct genotypes (*A_1_A_1_*, *B_1_B_1_* and *A_1_B_1_*) for exon 1 and eight distinct genotypes (*A_4_A_4_*, *B_4_B_4_*, *C_4_C_4_*, *D_4_D_4_*, *A_4_B_4_*, *A_4_C_4_*, *B_4_C_4_* and *A_4_E_4_*) for exon 4 were detected from the 290 piglet samples, while only one unique genotype (*A_3_A_3_*) was identified in exon 3 region (Figure 1A). After sequencing, two and five alleles were identified in exons 1 and 4, respectively. After aligning all allelic sequences of *DRA* exons in the Immuno Polymorphism Database (IPD)-MHC SLA and Genbank databases, *B_4_* in exon 4 was confirmed as a novel allele of *SLA-DRA* gene. All alleles of *DRA* gene exons identified in this study were submitted to the GenBank database; the obtained accession numbers are shown in .

In 290 Chinese Yantai black pigs, *A_1_* was detected at a frequency of 0.55 and *A_4_* at a frequency of 0.41, which were the most common variants for exon 1 and 4, respectively. The genotype frequencies of *SLA-DRA* gene exons disclosed a significant deviation in the assumption of Hardy–Weinberg equilibrium (HWE) (*p* < 0.01) (Table 2). The *oHet* and *eHet* based on allelic frequencies were 0.260, 0.495 in exon 1 and 0.439, 0.667 in exon 4, the *A_E_* was 1.979 and 3.087 in exon 1 and 4, respectively.

### 3.2. Gene Variations and Population Genetic Parameters of SLA-DRA Gene

Compared with the reference sequence (accession no: AY303990), nine SNPs across exons 1, 2 and 4 were detected in this population. Of these, two SNPs (c.178A > G and c.179G > A) were detected in exon 1 including one non-synonymous mutation, whereas seven SNPs (c.4167A > G, c.4185C > A, c.4196A > C, c.4205G > T, c.4208C > T, c.4246A > G and c.4293G > A) were identified in exon 4 containing two synonymous mutations (Figure 1B; Table 2).

**Figure 2:**
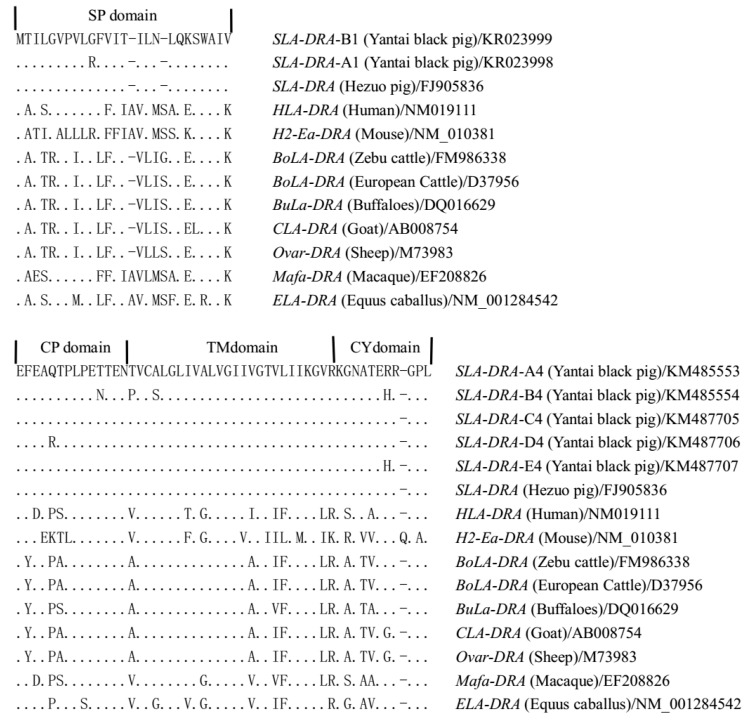
The alignment of the protein encoded by the *DRA* gene exon 1 and 4 among Yantai black pig and other vertebrates. The *DRA* genes were downloaded from pig (*S. tibetan*, FJ905836), humans (*H. sapiens*, NM019111), mice (*M. musculus*, NM010381), zebu cattle (*B. indicus*, FM986338), European cattle (*B. taurus*, D37956), buffaloes (*B. bubalis*, DQ016629), goat (*C. hircus*, AB008754), sheep (*O. aries*, M73983), macaque (*M. mulatta*, EF208826) and equus caballus (*E. caballus*, NM001284542). Exon 1 and 4 encoded the signal peptide, connecting peptide, transmembrance and cytoplasmic domains. A dot (.) indicates amino acid identity, and the hyphen (-) indicates a gap inserted to maximum alignment.

It was notable that c.179G > A substitution in exon 1 putatively resulted in a non-conservative amino acid change of p.Gln10Arg in the signal peptide (SP) domain. Exon 4 contained four transitions (c.4167A > G, c.4208C > T, c.4246A > G and c.4293G > A) and three transversions (c.4185C > A, c.4196A > C and c.4205G > T), which putatively resulted in the conservative amino acid changes (p.Gln206Arg, p.Thr212Asn, p.Thr216Pro, p.Ala219Ser and p.Arg248His) in the connecting peptide (CP), transmembrance (TM) and cytoplasmic (CY) domains (Figure 2). The conversion of an amino acid would contribute to the changes in function and structure of the corresponding protein.

The linkage disequilibrium analysis of SNPs in *DRA* gene exons (variations of *DRA* exon 2 are presented in Supplementary Materials: Table S1) indicated that the D’ values ranged from 0.028–0.167 and the r^2^ values were 0.000–0.007 (Supplementary Materials: Table S2), which both showed that these adjacent SNPs had little linkage disequilibrium. The possibility is that recombination will be high and LD will be low in genovariation-dense regions.

### 3.3. Amino Acid Alignments of the DRA Gene

The *SLA-DRA* gene of Yantai black pigs had the highest sequence similarity to the *DRA* gene of other swine breeds when compared to the respective *DRA* genes of pig, human, mice, zebu cattle, European cattle, buffaloes, goat, sheep, macaque and equus caballus. The alignments of amino acid sequences of vertebrate *DRA* genes are shown in Figure 2 (Supplementary Materials: Sequence S1 and Sequence S2). When the amino acid sequences at the *DRA* coding region of Yantai black pig and other swine breeds in GenBank database were aligned, there were only four amino acid differences in the region encoded by exon 1 and six amino acid differences in the region encoded by exon 4 at position 10, 16, 18, 22, 206, 212, 216, 219, 225 and 248 of the amino acid sequence, respectively (Supplementary Materials: Sequence S3 and Sequence S4).

### 3.4. Effects of SLA-DRA Gene Genotypes on Piglet Diarrhea

According to the statistical criterion that sample frequency of more than 2% is useful for disease-association analysis [38], the genotype *D_4_D_4_* in exon 4 was ruled out in linear contrast analysis due to a low frequency. The least square means (LSM) and standard errors (SE) of the observed diarrhea scores (correspond to Y_ijk_ values of the statistics model) among different genotypes of *SLA-DRA* gene exons 1 and 4 are presented in Table 3. Although no significant difference between genotypes of exon 1 and diarrhea (*p* > 0.05), the genotypic linear contrasts suggested that the diarrhea score of genotype *A_1_B_1_* (0.89 ± 0.14) was lower than that of genotypes *A_1_A_1_* (1.00 ± 0.11) and *B_1_B_1_* (1.08 ± 0.12). A significant association between genotypes of exon 4 and diarrhea was found, with the diarrhea scores of genotypes *C_4_C_4_* (1.80 ± 0.33) and *A_4_E_4_* (1.66 ± 0.25) being significantly higher than others (*p <* 0.01). The individuals of genotypes with higher diarrhea scores should be excluded in the artificial disease-resistant breeding, due to the higher incidence of diarrhea.

## 4. Discussion

Genetic diversity is essential for preserving adaptive potential of species and improving productivity of selected breeds. This study reports two alleles in *DRA* exon 1 and five alleles in *DRA* exon 4. This indicates that the *DRA* gene has much lower genetic diversity than SLA II *DQA, DQB* and *DRB* genes and other vertebrate MHC genes [12,39]. Moreover, the *oHet* value of exon 1 was lower than that of exon 4 and the *oHet* values in exon 1 and 4 loci were lower than those of *eHet* values, which is consistent with those reported in introduced pigs (Large White and Landrace) and domestic pigs (Bamei and Gansu black pig) [19,40]. This suggests that *DRA* is more homozygous and less polymorphic in exon 1 compared to exon 4. The result of *oHet* also indicates that Yantai black pigs are more vulnerable to artificial selection, migration, and genetic drift than domestic pigs, but less than introduced pigs. The low *oHet* may contribute to inbreeding and limiting genetic diversity. All loci were extremely deviated from the HWE (*p* *<* 0.01), implying that past selection had acted on the *DRA* gene in this population.

In Yantai black pigs, a total of seven alleles in *DRA* gene exons 1 and 4 were detected, which increased the numbers of *DRA* sequences 58 in pigs. Interestingly, we found that three SNPs (c.4185C > A, c.4196A > C and c.4205G > T) in exon 4 were strongly linked in this population and thus created a linkage of SNPs. Another strong link was found between nucleotide A of position 4293 and nucleotide G of position 4246. On the contrary, nucleotide G of position 4246 was not strongly linked with A of position 4293. Further studies are needed to investigate the structure and function of the linkage relationship.

Alignment of all the homologous sequences of swine *DRA* gene in GenBank reveals a total of thirty-three SNPs. Of these, eleven SNPs were identified in this study, four SNPs (c.4185C > A, c.4196A > C, c.4205G > T and c.4208C > T in exon 4) were the novel mutations, and the remaining SNPs have been reported in various of swine breeds, suggesting that these remaining SNPs are the common nucleotide variations and may play certain important roles in elementary characters and disease resistance. It is considered that most new mutants are derived from common variations, implying that rare variants represent the recent mutations [41]. The rare variants may be the results of adapting to the changing environment and facilitating the creation of new biological resources. The numbers of SNPs found in *DRA* are lower than other SLA genes, which is consistent with the limited diversity of *DRA* gene [42]. Furthermore, the number of SNPs associated with the *DRA* gene in Yantai black pigs was lower than that reported in introduced breeds [19]. It is suggested that local breeds have a more conservative *DRA* gene than introduced pigs. This conservation is the stable genetic foundation of the excellent characteristics and pivotal roles of the *DRA* gene, and may have certain correlation with providing protection in immune response.

The signal peptide (SP) and other functional domains including the α1, α2, connecting peptide, transmembrane and cytoplasmic domains are highly conserved, particularly in the sites associated with biological function in those *DRA* genes [43]. The SP domain has been considered to be the most variable region of the molecule across species [44]; however, a low level of variation was observed for the SP coding region of the *SLA-DRA* gene with only six SNPs resulting in four amino acid replacements (p.Gln10Arg, p.Leu16Ser, p.Leu18Ser and p.Trp22Arg) being identified in exon 1. Nine SNPs resulting in six amino acid replacements (p.Gln206Arg, p.Thr212Asn, p.Thr216Pro, p.Ala219Ser, p.Ala225Pro and p.Arg248His) had been identified in exon 4, which encoded the CP/TM/CY domain. *SLA-DRA* molecules showed high conservation and homology, which is consistent with the findings in other mammalian species [8,9]. The amino acid alignment indicated that the amino acid sequences of *DRA* genes from non-ruminant and ruminant (cattle and sheep) species had two different types of amino acid composition (Figure 2), which suggests that these species may have been split into different evolution patterns in natural selection. In addition, the amino acid numbers increase with the increasing classification status of animals, with human, mice, macaque and equus caballus having the longest amino acid sequences. Yantai black pig appears to be closest to the swine. The mutations in the coding region of the *DRA* gene may contribute to the antigen specificity [4], and the characteristics of MHC genes may provide information for a specific immune response to common or similar pathogens, which is in agreement with other groups, such as primates, cattle and sheep.

The well-characterized SLA alleles and genotypes have been shown to be associated with the resistances of immune-related diseases [44,45]. Exon 4 encodes transmembrane and cytoplasmic domains of the α chain antigen, which is responsible for transmembrane transport and localization [1,4]. In exon 4, genotype *C_4_C_4_* was found to have the highest diarrhea scores of 1.80 ± 0.33 in this study. Yang et al. reported that the *CE* genotype (where *C* is equal to *C_4_* in this study) was a susceptible genotype with a high piglet scores in Large White, Landrace and Duroc [19]. This suggests that allele *C_4_* may be susceptible to piglet diarrhea.

Heterozygotes are considered to have advantage over homozygotes in term of immune resistances, and many homozygotes of *DRA* have been reported to have higher diarrhea scores than heterozygotes, such as homozygotes *AA* and *BB* in exon 2, *AA* in exon 4 of *DRA* in Large White, Landrace and Duroc [19], and *BB* and *DD* in exon 4 of *DRA* gene in Bamei, Juema and Gansu black pigs [40]. These homozygous genotypes are considered as the probable susceptible genotypes to diarrhea. In contrast, some heterozygotes of SLA-*DQA* and *BoLA-DQA1* are resistant to diarrhea, mastitis and leukemia virus [18,46]. These findings suggest that heterozygous individuals of MHC are favorable in adaptive immunity against infectious diseases. However, heterozygote *A_4_E_4_* had a certain disease susceptibility with high piglet scores of 1.66 ± 0.25 in this study. It is speculated that homozygote *E_4_E_4_* may be more susceptible to piglet diarrhea. Unfortunately, no individuals with homozygote *E_4_E_4_* were detected in this study. The reason why homozygote *E_4_E_4_* genotypes are absent from this breed and its undefined association with diarrhea disease require further investigation.

## 5. Conclusions

We characterized the diversity of SLA α chain DRA gene in Yantai black pigs and the essential function to piglet diarrhea resistance or susceptibility, which could potentially impact on the porcine immune response and provide a rationale for observed variable immune responses to disease challenges. Future studies are warranted to investigate the linkage between swine immunogenetic diversity and pathogen community structure to better understand the underlying immunity mechanisms of infective diseases.

## Figures and Tables

**Table 1:** Primer sequences and SSCP condition of *SLA-DRA* gene exons.

Region	Location ^a^	Primer F/R(5′→3′)	Amplification Fragment	Annealing Temperature (°C)	SSCP Condition
Exon 1	151–226	F: CTTTGCTTGTATTGC R: ACCTAACTACCCCTC	186 bp	56.8	4 °C, 12%, 39:1, 190 V 20 h
Exon 3	4595–4876	F: TGCTAAACAGGGAAGGCT R: ACAAAGGAGACTGAGGGATG	352 bp	56.8	4 °C, 10%, 39:1, 200 V 20 h
Exon 4	4155–4309	F: TCCCGTAATACATCGTTC R: TTCCTTTCCTTGGCTCAT	357 bp	55.6	18 °C, 10%, 29:1, 200 V 18 h

^a^ Nucleotide positions refer to the *SLA-DRA* sequence in GenBank, accession No. AY 303990.

**Table 2:** Frequencies and genetic polymorphism parameters of *SLA-DRA* exons alleles in Yantai black pigs.

Locus	Allele	F (%) ^A^	GenBank Accession Number	*A_E_* ^B^	*oHet/eHet* ^C^	*PIC* ^D^	*χ^2 E^*	GenBank Ident 100%
								Accession Number/Breed
Exon 1	*A_1_*	0.55	KR023998	1.979	0.260/0.495	0.372	65.77 *	JX135565/Landrace
	*B_1_*	0.45	KR023999					LC002669/Hebao pig FJ905824/Gansu white pig
Exon 4	*A_4_*	0.41	KM485553	3.087	0.439/0.677	0.612	511.97 *	KP324812/Juema pig FJ905836/Hezuo pig
	*B_4_*	0.34	KM485554					Not found
	*C_4_*	0.20	KM487705					KP324810/Juema pig AY247781/Hebao pig
	*D_4_*	0.01	KM487706					KP324814/Juema pig AY191779/Banna minipig
	*E_4_*	0.04	KM487707					EU432071/Meishan pig EU722916/CMS minipig DQ883222/Korean native pig

^A^ F(%) = Frequency (%); ^B^ *A_E_* = number of effective alleles; ^C^ *oHet/eHet* = Observed/expected heterozygosity; ^D^ *PIC* = Polymorphism information content; ^E^ *χ****^2^*** = Chi-square test for Hardy-Weinberg equilibrium, * *p* < 0.01.

**Table 3:** Association between genotypes of *SLA-DRA* exons and piglet diarrhea in Yantai black pigs.

Exon 1							Exon 4						
Genotype	SN ^A^	Case	Control	OR ^B^	LSM ± SE ^C^	*p*	Genotype	SN	Case	Control	OR	LSM ± SE	*p*
*A_1_A_1_*	123	60	63	1.140	1.00 ± 0.11	0.679	*A_4_A_4_*	66	26	40	0.674	0.81 ± 0.14	0.302
*B_1_B_1_*	92	44	48	0.957	1.08 ± 0.12	0.424	*B_4_B_4_*	80	40	40	0.623	0.98 ± 0.13	0.462
*A_1_B_1_*	75	32	43	0.794	0.89 ± 0.14	0.348	*A_4_B_4_*	12	4	8	0.553	0.80 ± 0.33	0.114
							*C_4_C_4_*	12	8	4	2.344	1.80 ± 0.33	0.001
							*A_4_C_4_*	72	32	40	0.877	0.85 ± 0.14	0.933
							*B_4_C_4_*	22	8	14	0.625	0.77 ± 0.25	0.715
							*A_4_E_4_*	22	14	8	2.094	1.66 ± 0.25	0.002

^A^ SN = Sample Number; ^B^ OR = Odds Ratio; ^C^ LSM ± SE = The least squares means and standard errors of the observed piglet diarrhea scores, which are equivalent to the value Y_ijk_.

## Supplementary Materials

The following are available online at www.mdpi.com/2073-4425/7/7/36/s1, Table S1: Diversity and redefined names for the *SLA-DRA* gene exon 2 alleles described previously, Table S2: The linkage disequilibrium analysis of the *SLA-DRA* gene exon 1, 2 and 4 loci, Sequence S1: The amino acid alignments of *DRA* gene exon 1 among Yantai black pigs and other vertebrates, Sequence S2: The amino acid alignments of *DRA* gene exon 4 among Yantai black pigs and other vertebrates, Sequence S3: The amino acid alignments of *SLA-DRA* gene exon 1 among Yantai black pigs and other twenty-eight swine species in GenBank, Sequence S4: The amino acid alignments of *SLA-DRA* gene exon 4 among Yantai black pigs and other twenty-eight swine species in GenBank.
