# Supplementary material for: Effect of Genetic Diversity in Swine Leukocyte Antigen-DRA Gene on Piglet Diarrhea
Source: Genes (Basel). 2016 Jul 15;7(7):36. doi: 10.3390/genes7070036 (PMC4962006; doi:10.3390/genes7070036)
Supplement: Supplementary file 1 [file genes-07-00036-s001.zip › Supplementary file Tables-final-.docx]

**Supplementary Materials: Effect of Genetic Diversity in Swine Leukocyte Antigen-*DRA* Gene on Piglet Diarrhea**

Xiaoyu Huang, Qiaoli Yang, Junhu Yuan, Lixia Liu, Wenyang Sun, Yingdi Jiang, Shengguo Zhao, Shengwei Zhang, Wangzhou Huang and Shuangbao Gun

**Table S1.** Diversity and redefined names for the *SLA-DRA* gene exon 2 alleles described previously.

| **Allele Name** | | **Mutation Site** | **GenBank Accession Number** | **Reference** |
| --- | --- | --- | --- | --- |
| Redesign | Original |  |  |  |
| *A_2_* | A |  | KM411976 | Yang et al., 2015 [1] |
| *B_2_* | B | c.3093A > C | KM411975 |  |
| *C_2_* | C | c.3104C > T | KM411978 |  |

Note: The reference sequence was the accession no.AY303990 in GenBank database.

**Table S2.** The linkage disequilibrium analysis of the *SLA-DRA* gene exon 1, 2 and 4 loci.

| **LD parameters** | **Exon 1-Exon 2** | **Exon 1-Exon 4** | **Exon 2-Exon 4** |
| --- | --- | --- | --- |
| r^2^ | 0.001 | 0.000 | 0.007 |
| D′ | 0.047 | 0.028 | 0.167 |

**Reference**

1. Yang, Q.L.; Huang, X.Y.; Yuan, J.H.; Gun, S.B. Polymorphisms in the SLA-DQA and DRA gene exon2 and their association with piglet diarrhea in Chinese Yantai black pig. *Philipp. Agric. Sci*. **2015**, *98*, 253–261.
